# Inadequate Adenosine-Induced Flow Arrest During Intraoperative Basilar Aneurysm Surgery

**DOI:** 10.7759/cureus.42239

**Published:** 2023-07-21

**Authors:** Ana Pereira, Sara Salvador, Helena Sousa, Manuela Casal

**Affiliations:** 1 Anaesthesiology, Hospital Vila Franca de Xira, Lisbon, PRT; 2 Anaesthesiology, Centro Hospitalar Universitário do Porto, Porto, PRT; 3 Anaesthesiology, Centro Hospitalar do Baixo Vouga, Aveiro, PRT

**Keywords:** neuroanesthesiology, adenosine, cardiac standstill, surgical clipping, ruptured cerebral aneurysm

## Abstract

Aneurysmal subarachnoid hemorrhage (SAH) is an acute neurologic emergency. We report the case of a 48-year-old male with a massive SAH caused by a ruptured aneurysm of the vertebrobasilar transition. During an urgent craniotomy, due to an aneurysm re-rupture, adenosine was given for flow arrest but no sinus pause was observed. Esmolol was administered and strategies for cerebral protection were implemented. The surgeon was able to clip the aneurysm and the patient was discharged after 78 days without sequelae. The highest adenosine dose given did not result in an efficient cardiac pause. Atropine given one hour before could have contributed to this. This case highlights a successfully managed case of ruptured aneurysm with refractory adenosine-induced flow arrest.

## Introduction

The overall incidence of spontaneous subarachnoid hemorrhage (SAH) is about 6.1 per 100,000 cases per year. About 85% of cases are caused by intracranial aneurysm rupture [1]. Posterior circulation aneurysms involving the vertebrobasilar system, although rare, have the highest rates of rupture [2].

Patients are usually females under 55 years old, with hypertension, hypercholesterolemia, cigarette smoking, and family history accounting for risk factors. Most survivors of SAH have long-term disability or cognitive impairment [1].

Endovascular coiling or surgical repair are the only effective treatments for ruptured aneurysms and should be performed as early as possible to prevent further morbidity and rebleeding [3].

The anesthetic management for craniotomy in aneurysmal SAH can be quite challenging. Perioperative care should focus on facilitating early definitive treatment and surgical exposure while maintaining cerebral perfusion, preventing brain swelling, as well as managing crises (aneurysm rupture) [4].

Temporary clipping on parent vessels by reducing blood flow facilitates dissection and enhances accurate placement of the permanent clipping while reducing the risk of rupture. Sometimes the aneurysm anatomy is not favorable for temporary clipping and adenosine can be used for a temporary flow arrest, which can also be used during intraoperative rupture [4]. Adenosine causes sinus bradycardia, followed by a pause and cardiac arrest, reducing cardiac output and mean arterial pressure (MAP), and, in turn, reducing the hemorrhage and improving conditions for clipping a cerebral aneurysm.

Here, we present the management of a ruptured basilar aneurysm with massive intraoperative rebleeding where it was difficult to define the adequate doses of adenosine to perform an adenosine-induced flow arrest. Reports of the ideal dose of adenosine-induced flow arrest are lacking in the literature, and it is yet to be defined clearly.

## Case presentation

**Figure 1:**
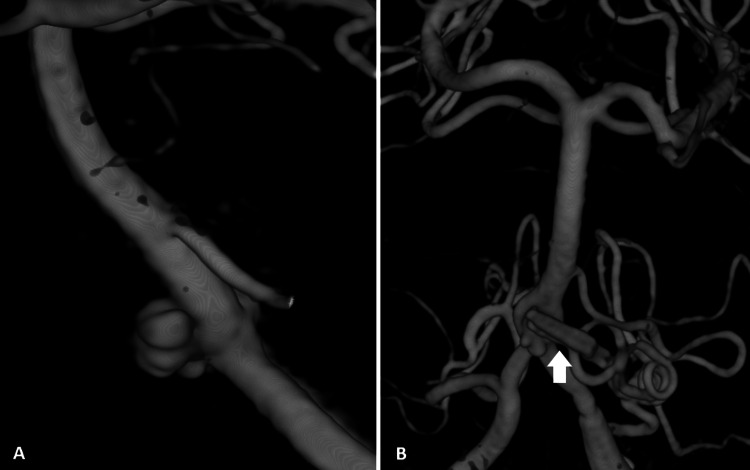
Three-dimensional cerebral digital angiography showing a basilar aneurysm before and after surgical clipping. A: Three-dimensional cerebral digital angiography showing pre-clipping basilar aneurysm. B: After surgical clipping of the basilar aneurysm (arrow: clip).

A 48-year-old male (weighing 75 kg), without relevant medical history, suddenly developed an intense and diffuse headache with associated vomiting. He was initially evaluated in the Emergency Department presenting with altered neurological status with spontaneous eye opening, roving eyes, and aphasia. Pupils were isochoric but slightly photoreactive. No initial motor or sensitive deficits were evident. On initial evaluation, he presented with sinus bradycardia (58 beats/minute) and mild hypertension (systolic blood pressure of 150 mmHg). Due to the rapidly deteriorating Glasgow Coma Scale score to 10 (O3V1M6), the airway was secured with tracheal intubation. Initial computerized tomography scan showed a massive SAH with intraventricular bleeding (Fisher IV) compatible with cerebral aneurysm rupture from the vertebrobasilar transition (Figure 1, Panel A). His initial laboratory workup was unremarkable. The patient was then transferred to a tertiary center for definitive treatment.

An external ventricular drain was placed and intracranial pressure was monitored (persistently under 20 mmHg). A first attempt was made to manage the aneurysm by endovascular coiling, but it was not possible. Hence, the patient was scheduled for surgical clipping. Blood typing, crossmatching, and reservation of 4 red blood cell (RBC) units were verified and anesthetic consent was presumed.

The patient was transferred to the operating room 30 hours post-ictus, sedated with propofol and remifentanil (Richmond Agitation Sedation Scale: 5), under controlled ventilation (SpO_2_: 99%, ETCO_2_: 35 mmHg), with MAP of 88 mmHg, sinus rhythm at 65 beats/minute, and ventricular drainage at 16 mmHg.

The American Society of Anesthesiologists standard monitoring was complemented with processed electroencephalography, regional cerebral oximetry, and neuromuscular block monitoring. As right subclavian central venous (CVC) and radial arterial catheters were already in place, remifentanil and propofol infusions were used according to target-controlled infusion. A craniotomy was performed in park bench positioning.

**Figure 2:**
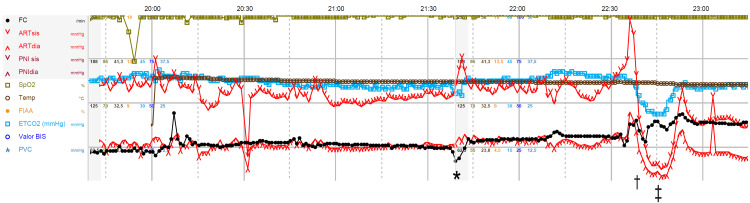
Intraoperative monitoring. *: atropine administration; †: adenosine administration; ‡: placement of the definite clip on the aneurysm neck

During dura anchoring, the patient developed severe bradycardia with sinus pause reverted with 1 mg of intravenous (IV) atropine (Figure 2).

Afterward, the patient remained hemodynamically stable until one hour later, when during an attempt at temporary clipping, an aneurysm rupture occurred. At first, the patient became hypertensive (blood pressure: 220/96 mmHg). An adenosine-induced flow arrest was attempted with a total of 42 mg (6 mg, 12 mg, 24 mg) followed by a rapid flush through the CVC, without any resulting asystole period. The flow arrest was then tried with esmolol (0.8 mg to 1 kg). Other strategies for cerebral protection were simultaneously implemented such as mild hyperventilation, osmotic therapy with hypertonic 8% saline, intraventricular drainage, and induced burst suppression with thiopental (5 mg to 1 kg IV). These strategies were titrated to neuromonitoring and serial blood gas analysis. The surgeon was able to place the definite clip on the aneurysm’s neck (Figure 1, Panel B). However, due to massive blood loss estimated at 2,000 mL (hemoglobin variation of 5.1 d/dL), the patient evolved with hypovolemic shock. Vasopressor support was initiated with noradrenaline (maximum 0.49 µg/kg/minute) and fluid was replaced with 1,500 mL of a balanced crystalloid solution and 3 units of RBCs. Calcium was also supplemented.

By the end of the procedure, the patient was hemodynamically stable without vasopressor support (MAP> 65 mmHg and bilateral near-infrared spectroscopy >70), and he was transferred to the intensive care unit, sedated and ventilated.

Postoperatively, it was possible to progressively reduce vasopressors with shock resolution. Some complications occurred afterward such as severe diffuse vasospasm, which was treated with milrinone and intra-arterial verapamil, and subacute communicating hydrocephalus, which required a ventriculoperitoneal shunt. After extubation, lower cranial nerve dysfunctions (dysphonia and dysphagia) were identified, for which he underwent intensive rehabilitation. The patient was discharged on the 78th post-ictus day with a normal neurological evaluation.

## Discussion

It has been 92 years since the first ruptured cerebral aneurysm surgery. For a long time, anesthesiologists and neurosurgeons have strived to find the best methodology to manage intraoperative rupture. Induced hypothermia, cardiopulmonary bypass, pharmacologically induced hypotension, and cardiac standstill are some of the methodologies historically used to address the issue [5]. In selected patients and adjusting the extent and duration, controlled hypotension not only allows the reduction of aneurysmal wall tension and the risk of rupture but also decreases the probability of bleeding and improves conditions for successful clipping [6].

Adenosine is a naturally occurring nucleoside that binds to G-protein-coupled A1 receptors, initiating a cascade through activation of adenylyl cyclase, decreasing intracellular cyclic AMP and suppressing calcium-dependent action potentials, which results in depressed nodal conduction and decreased atrial contractility and ventricular automaticity. It also activates an inward potassium current resulting in hyperpolarization. Following rapid administration, adenosine causes sinus bradycardia, followed by a pause and cardiac arrest, reducing cardiac output and MAP. It is promptly metabolized inside RBCs, with a half-life of only 0.6-20 seconds. Therefore, its cardiovascular effects are transient, which makes it an ideal drug for induced flow arrest [6,7].

In 1999, Groff et al. published the first case report about adenosine-induced cardiac standstill with complete flow arrest for the clipping of a cerebral aneurysm. The circulatory arrest induced by adenosine did not seem to cause significant negative outcomes, with clear benefits during intraoperative management of sudden aneurysm rupture and uncontrollable bleeding [5].

Guidelines concerning the management of aneurysmal SAH mention the adenosine-induced transient cardiac pause as an intervention for decompressing large aneurysms or controlling intraoperative hemorrhage; however, controlled studies are needed to validate this intervention. [3].

In this case, the patient presented with a basilar aneurysm that had a first rupture, and then rebled intraoperatively with massive blood loss. As stopping the hemorrhage and aneurysm clipping was the priority, circulatory flow arrest was attempted with IV bolus adenosine; however, no sinus pause or hypotension occurred. Several questions were raised to explain its inefficacy: was adenosine dose-weight appropriate? Did the previously administered atropine affect the adenosine mechanism of action?

In this case, for flow arrest, adenosine was administered at an initial dose of 6 mg (0.08 mg/kg), followed by 12 mg (0.16 mg/kg), and then 24mg (0.32 mg/kg), always followed by a rapid saline IV flush. Nonetheless, not even the highest adenosine dose resulted in efficient cardiac pause or temporary hypotension.

To date, there is a lack of data concerning the ideal dose of adenosine for flow arrest, and specific guidelines have not addressed this issue [6]. Hashimoto et al. used adenosine in endovascular embolization of cerebral arteriovenous malformations. The initial dose was 0.25-0.35 mg/kg with an extra 10-20 mg bolus until targeted hypotension (MAP: 25-30 mmHg). The duration of hypotension correlated linearly with the adenosine dose, with 0.98 ± 0.40 mg/kg being the dose needed for sustained asystole and hypotension [8].

Bebawy et al. reviewed 24 aneurysm clip ligation to demonstrate a dose-response curve for adenosine. A median dose of 0.34 mg/kg (range: 0.29-0.44 mg/kg) resulted in sustained hypotension (systolic blood pressure <60 mmHg) for a median of 57 seconds. It is important to mention that all patients were receiving 0.5 MAC volatile anesthetic, 0.1 µg/kg/minute remifentanil, and 50-150 µg/kg/minute propofol for a burst suppression rate of 0.7-0.8 at the time of aneurysm clipping. Based on the data, the authors recommend a starting dose of 0.3 to 0.4 mg/kg adenosine to achieve about 45 seconds of profound systemic hypotension [9]. In this case, the patient was under a target-controlled infusion IV anesthesia with propofol and remifentanil, adjusted according to processed electroencephalography (Bispectral Index™ between 40-60 [Medtronic, Minneapolis, United States of America]) with no burst suppression registered during aneurysm rupture or adenosine administration. Thus, despite the latest adenosine dose being within the recommended range, the anesthesia depth was not as endorsed by Bebawy et al. which may have contributed to its inefficacy.

Another mechanism we contemplated in our discussion was the previous administration of atropine. During dura manipulation, the patient developed severe bradycardia with a sinus pause which was reverted with 1 mg of atropine. About an hour after, adenosine was administered for flow arrest. Atropine is a muscarinic antagonist that works through the inhibition of postganglionic acetylcholine (ACh) receptors primarily located in the heart. In a standard dose, such as 1 mg for an adult, atropine blocks ACh action in the sinoatrial node, inhibiting nodal cell hyperpolarization, the opposite mechanism as adenosine. Atropine is widely distributed in the body, with its elimination occurring in two phases: a rapid phase of two hours, followed by a slow phase of 13 hours. This means that when adenosine was administered, atropine was still in circulation, which can be another factor responsible for its refractoriness [7].

Adenosine is also less effective in the presence of adenosine receptor blockers such as theophylline [7]; however, this cause can be ruled out as the patient was not under any methylxanthine.

In similar cases, Nimjee et al. described a patient with an aneurysm from the anterior circulation who received a total of 744 mg of adenosine, administered in 11 divided doses, with a maximal dose of 90 mg, resulting in successful clipping. The authors did not reach a conclusion regarding the cause of resistance wondering if it would be a genetic variation from the adenosine receptor, downstream signaling, or some inter-individual resistance to purine nucleoside medications [10].

Despite the non-efficacy of adenosine, the use of betablockers and induced burst suppression with thiopental likely allowed temporary hypotension and successful clip placement. Thiopental by decreasing cerebral metabolic rate of oxygen during this short period as well as other cerebral protection strategies contributed to the good neurological outcome. After a long recovery and rehabilitation process, the patient was discharged fully autonomous in his daily activities.

## Conclusions

More studies addressing the ideal dose of adenosine for flow arrest are needed. The adenosine dose given in this case seems to be within the range used, but it did not have the expected result. The effect of atropine on the heart conduction system could have contributed to it.
